# Math Is for Me: A Field Intervention to Strengthen Math Self-Concepts in Spanish-Speaking 3rd Grade Children

**DOI:** 10.3389/fpsyg.2020.593995

**Published:** 2020-11-23

**Authors:** Dario Cvencek, Jesús Paz-Albo, Allison Master, Cristina V. Herranz Llácer, Aránzazu Hervás-Escobar, Andrew N. Meltzoff

**Affiliations:** ^1^Institute for Learning and Brain Sciences, University of Washington, Seattle, WA, United States; ^2^Departamento de Ciencias de la Educación, Lenguaje, Cultura y Artes, Ciencias Histórico-Jurídicas y Humanísticas y Lenguas Modernas, Facultad de Ciencias Jurídicas y Sociales, Universidad Rey Juan Carlos, Madrid, Spain; ^3^Department of Psychological, Health, and Learning Sciences, University of Houston, Houston, TX, United States

**Keywords:** math self-concept, intervention, implicit cognition, math achievement, Spanish-speaking children, elementary school

## Abstract

Children’s math self-concepts—their beliefs about themselves and math—are important for teachers, parents, and students, because they are linked to academic motivation, choices, and outcomes. There have been several attempts at improving math achievement based on the training of math skills. Here we took a complementary approach and conducted an intervention study to boost children’s math self-concepts. Our primary objective was to assess the feasibility of whether a novel multicomponent intervention—one that combines explicit and implicit approaches to help children form more positive beliefs linking themselves and math—can be administered in an authentic school setting. The intervention was conducted in Spain, a country in which math achievement is below the average of other OECD countries. We tested third grade students (*N* = 180; *M*_*age*_ = 8.79 years; 96 girls), using treatment and comparison groups and pre- and posttest assessments. A novelty of this study is that we used both implicit and explicit measures of children’s math self-concepts. For a subsample of students, we also obtained an assessment of year-end math achievement. Math self-concepts in the treatment and comparison groups did not significantly differ at pretest. Students in the treatment group demonstrated a significant increase in math self-concepts from pretest to posttest; students in the comparison group did not. In the treatment group, implicit math self-concepts at posttest were associated with higher year-end math achievement, assessed approximately 3 months after the completion of the intervention. Taken together, the results suggest that math self-concepts are malleable and that social–cognitive interventions can boost children’s beliefs about themselves and math. Based on the favorable results of this feasibility study, it is appropriate to formally test this novel multicomponent approach for improving math self-concepts using randomized controlled trial (RCT) design.

## Introduction

Improving the quality of education during the elementary-school years is a goal of global initiatives concerned with transforming schools. There has been increasing attention to enhancing not only children’s academic skills, but also students’ beliefs and attitudes about school and learning. Research has shown that students’ thoughts and feelings about mathematics contribute to their academic motivation, choices, and achievement. The present study examined the malleability of students’ *math self-concepts*—how children think of themselves in relation to mathematics. We designed an intervention to enhance children’s math self-concepts with the long-term goal, after further study, of designing broader intervention programs to help improve mathematics outcomes in young children.

At the broadest level, math self-concepts refer to how children think of themselves in relation to math. A substantive body of empirical work in the traditions of Reciprocal Effects Model (REM; Marsh, 1990) and Expectancy Value Theory (EVT; Eccles et al., 1983) provides ample evidence that the relation between a self-concept in a particular subject (e.g., math self-concept) and achievement in that subject (e.g., math achievement) is positive and often reciprocal (see Arens et al., 2020; Jiang et al., 2020). For example, a large, longitudinal study from the end of Grade 4 through the end of Grade 9 (*N* = 3,370 German students across 42 schools) found that math self-concepts were both predictive of (as well as predicted by) math test scores and school grades over the 6-year-period (Marsh et al., 2018). Another study involving 241 Shanghai children from Grades 2, 4, and 7 showed, using path analyses, that math self-concepts were positively related to calculation fluency in Grade 7 and math problem solving in Grades 4 and 7 (Cai et al., 2018). Finally, a study of United States preschoolers showed that early math self-concepts predicted math achievement 5 months later, controlling for initial self-concepts/interest in math (Fisher et al., 2012). Taken together, these studies show that the links between math self-concepts and math achievement are robust, reciprocal, evident cross-sectionally and longitudinally, and across different countries and age groups.

Math self-concepts can be measured in many ways and at many levels (Gunderson et al., 2012). At the simpler level are straightforward self-representations and identities such as students’ judgments of their own personal ability in math (Harter, 2006) or a strong psychological link between *self* and *math* (Cvencek et al., 2011). At higher levels of complexity are multidimensional, self-reflective views of math self-concepts that involve social comparison, perceptions of the self in math learning situations, and future expectancies about one’s competence in math (Bong and Skaalvik, 2003; Eccles, 2005; Marsh et al., 2019). The identity association of *me* = *math* corresponds to the “simpler level” of the math self-concept, and is what we sought to tap in the tests used in this paper (Supplementary Material Section 1 provides a more detailed analysis of this conception of “levels,” as well as measurement methods).

Young children’s math self-concepts have been assessed with both *explicit* measures using intentional, verbalizable self-report (e.g., Harter and Pike, 1984), as well as *implicit* measures using automatic and non-reflective responding (Cvencek et al., 2011). Explicit processes are controlled and deliberative with mental contents accessible to introspection. Implicit processes are typically fast, non-deliberative, and not available to introspection. Both are recognized to be of psychological importance (e.g., Kahneman, 2011). Some studies have shown that, although implicit and explicit math self-concepts can be dissociated in children, both are useful for predicting math-related outcomes. Explicit math self-concepts may be more strongly linked to children’s conscious choices and future aspirations, while implicit math self-concepts may be more strongly linked to achievement on timed, high-stakes standardized tests (Steffens et al., 2010; Cvencek et al., 2015).

In research with adult participants, the explicit system is considered to be malleable and changed with “one-shot” intervention strategies, whereas the implicit system is often considered to be relatively rigid (Devine et al., 2012). To date, little research has examined whether the difficulties in changing implicit cognition in *adult* participants also apply for children. Interventions on children could potentially be more effective in changing implicit beliefs than in adults because children’s implicit cognition is based on fewer experiences and therefore may be less crystallized and more malleable than the adult case (Gonzalez et al., 2017). It is currently unknown whether interventions targeting implicit math beliefs can be designed in age-appropriate ways during elementary school, or whether a combination of interventions that draw on both implicit and explicit measurement approaches might be especially effective.

Elementary school is a desirable time for designing interventions to change math self-concepts. First, math self-concepts are still developing during this age period. While there is a substantial body of work demonstrating the stable relations between math self-concepts and math achievement by middle school (Muenks et al., 2018; Marsh et al., 2019), the math self-concepts in elementary-school children undergo substantial change (Ehm et al., 2019). Specifically, the magnitude (how positive or negative one’s math self-concept is), structure (what types of beliefs and self-evaluations factor into the “content” of one’s math self-concept), and the relation of math self-concepts to math achievement all undergo change during elementary school (Weidinger et al., 2018). Second, the ages tested here may represent the optimal time to influence children’s implicit beliefs in particular (Gonzalez et al., 2017; Qian et al., 2019); it may be a time during which math begins to be incorporated into one’s self-concept (Lei et al., 2019). The age group in this study (Grade 3; 8 to 9 years of age) was chosen based on dual reasons: (i) it seems to be a time of developmental change and (ii) previous findings show that math self-concepts can be measured reliably with both implicit and explicit measures at this age (Meltzoff and Cvencek, 2019).

To our knowledge, only one study attempted to intervene on both math achievement, as well as children’s thoughts and feelings about mathematics (math self-concept, math anxiety, and self-regulation) in elementary-school children (Collingwood and Dewey, 2018). This multi-component intervention was delivered by trained teaching assistants in small groups for 4 weeks and consisted of 12 45-minute long sessions. The intervention involved: (i) self-regulated learning, (ii) mindful breathing, (iii) humor and comic strips, and (iv) use of self-coping statements when solving math problems. The intervention was found to improve math achievement and self-regulation, but had no measurable effect on math self-concept or math anxiety. It is currently unknown how to effectively enhance students’ math self-concepts during elementary-school years.

The interventions used in the current study consisted of multiple components. The use of multi-component interventions is considered desirable in this age group, particularly when trying to establish larger effect sizes for constructs (such as math self-concepts) which are multi-dimensional themselves (Martin, 2008; and see Supplementary Material, Section 1). We designed and used age-appropriate interventions on *both* explicit and implicit self-concepts: The two “explicit” interventions required children to process information and engage in reflective thinking; the two “implicit” interventions allowed children to process information in a less deliberate manner and engage in automatic responding. A novel feature of the current work is that we combined *both* types of strategies in a single intervention session, because we believe that this increases the likelihood of success, as opposed to focusing on one type of approach alone.

The interventions targeting explicit cognitions drew on previous research showing that students benefit from feeling that they are valued and can succeed in school. It may be important for students to have these feelings both as individuals and in relation to the social-identity groupings to which they feel a sense of belonging (such as race/ethnicity, gender, or regional identity). In one previous study, children were most successful on a math test when they were reminded of a social identity that was linked to positive stereotypes in math, such as being Asian (Ambady et al., 2001). In another study, middle-school students of color achieved higher grades when their sense of personal adequacy was affirmed in school (Cohen et al., 2006). If children feel that they and their groups are successful in math, this should boost their explicit math self-concepts (Master and Meltzoff, 2020).

The interventions targeting implicit cognitions drew on previous successful interventions used in adults showing that mental associations between “me” and certain attributes can be strengthened using motor acts and auditory cues. In one study (Kawakami et al., 2008), college women who were low in implicit math identification (defined as the “strength of association between *self* and *math* versus *other* and *math*,” p. 821) showed greater math identification and persistence after a training that involved pulling a joystick toward themselves when they viewed images related to math. In another study, adults heard particular sounds after attending and responding to counter-stereotypical pairings between images and words, such as a female face with the word “math” (Hu et al., 2015). These sound cues were designed to reinforce the counter-stereotypical pairings in contrast to stereotypical pairings. We reasoned that in the case of children, similar physical and audio procedures could help reinforce the link between *me* and *math*, boosting children’s implicit math self-concepts.

The primary goal of this study was to assess the *feasibility* of whether a novel multicomponent intervention that combines explicit and implicit approaches to help children form more positive beliefs about themselves and math can be administered in an authentic school setting. Implementation research in education typically begins with an exploration of malleable factors that provides the initial empirical basis for refining a particular intervention. An overarching goal during this phase of research is to determine: (i) whether there is evidence of the promise of the intervention for achieving its intended outcomes, and (ii) whether the theorized intervention approaches are feasible for use (e.g., not too time consuming) within the intended authentic delivery setting (Institute of Education Sciences, 2012). Evidence of promise at this phase will usually lead to further research using randomized controlled trial (RCT) design to provide rigorous experimental data about the efficacy of the intervention.

Following this general model, we used a repeated-measures, quasi-experimental design to establish feasibility and provide guidance for future designs. We acknowledge that the authentic school settings imposed certain limitations on the study, which can, and should, be improved in future work. These issues are articulated and addressed in the section “Limitations, Lessons Learned, and Future Research.” At the same time, we think that this study, which is a first attempt at combining both implicit and explicit measures in an intervention on math self-concepts at this early age, advances our knowledge with potential downstream benefits in the design of broader educational interventions.

This study involved an international consortium of researchers from the United States and Spain and took place in Madrid. We chose Madrid as the test site for several reasons. First, the math achievement of students from Spain, as measured by PISA, is below the OECD average (Organisation for Economic Co-operation and Development, 2015). This below-average achievement provides an opportunity for testing new interventions because a successful intervention, after sufficient instrument development, may be able to be used in this same setting to enhance students’ math outcomes before students fall behind on standardized math achievement on international tests. Second, Madrid is the only region in Spain that makes each school’s average results on standardized tests available to the public (Anghel et al., 2015); and there is intense governmental and educational interest in boosting math performance in Spain in general and Madrid in particular. Indeed, we received considerable assistance from local policymakers, principals, and teachers in the conduct of this study. Third, by adapting the interventions for use in Madrid, this research has potential to provide tools in the Spanish language that can contribute to educational research not only in Europe but also in Latin America and an increasingly large Spanish-speaking population in the United States (Rivas-Drake et al., 2016; Bauman, 2017).

This study makes four novel contributions to the literature. First, to our knowledge, no previous study has examined the effectiveness of a multicomponent intervention targeting students’ math self-concepts in early elementary school. Second, this study was the first to use both implicit and explicit math self-concepts as outcome measures in elementary school, and to combine these psychological constructs to predict year-end math achievement. Third, no previous study has used technology-based interventions to influence children’s math self-concepts, which provides preliminary groundwork toward broadly-useable interventions. Fourth, the current study responds to recent calls for reducing the oversampling of North American participants in educational research (Nielsen et al., 2017).

## Materials and Methods

### Participants

The participants were 180 students (*M* = 8.79 years, *SD* = 0.40, range: 7.98–10.02 years); 96 were girls (*M* = 8.79 years, *SD* = 0.38, range: 8.04–9.95 years), and 84 were boys (*M* = 8.79 years, *SD* = 0.42, range: 7.98–10.02 years). Mean ages between girls and boys did not differ, *p* = 0.99. These participants were recruited from nine Madrid elementary schools (30 classrooms), with the cooperation of the Madrid Ministry of Education. The research team obtained permission from the school principals. Students were tested at schools either from (i) December to April during the 2015–2016 school year, or (ii) March to April during the 2016–2017 school year.

All nine schools shared the same regional department of education and educational policies, however, each school was free to design its own educational mission. All nine schools belonged to the same City of Madrid school district. On average, children attending the nine schools were primarily low- to upper-middle SES, but we did not specifically ask children about their individual family’s SES. None of the classrooms were special education classrooms. Finally, according to the official results of the external assessment of all Madrid Grade 3 students (1,302 schools) in May 2016, the City of Madrid school district had the average score of 7.08 for the Math Assessment (on a scale from 0 to 10; 549 participating schools total), indicating that the nine schools were medium-achieving in math.

Due to institutional constraints in some of the schools, students could not be fully randomized into the treatment and control groups; thus, our study was what Shadish et al. (2002) have described as a quasi-experiment. Quasi-experiments done in real-world settings can be a very useful step toward more randomized controlled designs (Cook et al., 2020). Specifically, Shadish et al. (2002) argue that “the use of carefully selected comparison groups facilitates causal inference from quasi experiments (when) they are also accompanied by pretest measures on the same outcome variable as posttest” (p. 136). In line with this reasoning, students in the present study were assigned to treatment and comparison groups. As recommended by Shadish et al. (2002), we used a pretest/posttest design and used the same outcome variables in the pretest and posttest. As will be shown, the treatment and comparison groups did not differ in their pretest scores for either the implicit or the explicit measure. Finally, we oversampled for the treatment group (see Berkowitz et al., 2015, for a similar strategy).

The research team consisted of native Spanish-speaking researchers from the Universidad Rey Juan Carlos, which is located in proximity to the schools. The team members visited schools and held informational meetings to explain the study goals, procedures, and resolve any questions regarding the educational interventions. Families gave written consent for their children. The procedures of this research were approved by the relevant university Research Ethics Committee (Universidad Rey Juan Carlos approval numbers: 22/2015 and ENM 22/20150712201600317).

### Materials

Students were tested individually in a separate room outside of his or her classroom by trained experimenters. For each measure, the student sat at a table facing a Lenovo ThinkPad Yoga 15 Ultrabook laptop computer with a pair of QuietComfort 25 Acoustic Noise Canceling headphones and an adapted keyboard (see Cvencek et al., 2015, pp. 3-4, for an illustration of such computerized implicit and explicit measures in educational research). The experimenter was seated next to the student and gave instructions orally. Each test session began with a 3–5-minute description of the study, during which students were told that they would “play a game on the computer” and were familiarized with the test apparatus. Students completed implicit and explicit tests of math self-concepts. No other tests pertaining to different school subjects other than the ones reported here were administered. The main characteristics of these tests are described in the sections below.

### Pretest

#### Implicit Measure

The implicit measure was a Child Implicit Association Test (ChIAT) that has been successfully used with this age to measure math stereotypes and self-concepts (Cvencek et al., 2011). The underlying principle of the ChIAT is that it is easier to give the same response to items that are associated in memory (called “congruent”) than to give the same response to pairs of items that are not associated in memory (“incongruent”). An example can illuminate this and help explain the general principles of the ChIAT. Imagine being presented with images of *spring* landscapes, *winter* landscapes, and also with faces of *young* people and faces of *old* people. You are asked to sort these images into two piles: one pile in which you are to place *spring* landscapes and *young* faces, and another pile in which you are instructed to place *winter* landscapes and *old* faces. Under these instructions, you will likely be very fast sorting the images: Your ease of sorting will be facilitated by a prior association of “spring goes with youth” and “winter goes with old age.” However, if you are asked to sort the same images again, but now you have to place *spring* landscapes and *old* faces in one pile, and *winter* landscapes and *young* faces in the other pile, it will likely be more difficult. This is because you probably do not have memory links between “spring goes with old” or “winter goes with young.” The underlying principle of the ChIAT is similar, and it has been found that both adults and children find certain associations to be more congruent, and they respond to them faster (which can be measured precisely on a computer machine). If children identify with math, they are expected to respond more quickly to *me* = *math* than to other control pairings (see Cvencek et al., 2011, for more complete details).

The math self-concept ChIAT assesses the degree to which individual participants link *me* with *math* more than with a different academic subject such as reading. During the math self-concept ChIAT, students sorted the words belonging to four different categories: *me*, *not-me*, *math*, and *reading*. All stimuli were presented in Spanish. The stimuli for the *me* and *not-me* categories were four *me* pronouns (me, myself, I, mine) and four *not-me* pronouns (they, them, theirs, other). The stimuli for the *math* and *reading* categories were five *math* words (addition, count, math, graph, numbers) and five *reading* words (books, letters, read, sentence, story).

The ChIAT was scored using the *D*-score algorithm, which converts the raw response times into a standardized metric of association strength in line with previous successful uses with elementary-school children (Baron and Banaji, 2006; Cvencek et al., 2011). The ChIAT score (*D*) was scored so it had computational upper and lower bounds of +2 (which indicated a strong association of *me* = *math*) to −2 (which was a strong association of *me* = *reading*), with a rational value of 0 indicating an equally strong association of *me* with *math* and *reading*. The ChIAT score provides a continuous measure, good internal consistency, and exhibits great variability in responses across different participants, which allows educational researchers to assess stronger or weaker identification with math on an interval scale that is highly sensitive to individual differences. In the current study, the implicit measure was internally consistent (α = 0.70). Using standard algorithms and procedures for eliminations for this age (Cvencek et al., 2011), four students (2.2%) were excluded for having excessively slow responses, and one (0.6%) was excluded for excessive errors, leaving *N* = 175 (94 girls, 81 boys) in the final reported sample.

#### Explicit Measure

The self-report measure was administered as two Likert-scale questions from the “Pictorial Scale of Perceived Competence and Acceptance for Young Children” (Harter and Pike, 1984). This measure used both pictures and verbal explanations. In the double binary response strategy used in the original scale, the experimenter first asked the student to select which of two same-gender characters, who were either engaged in math or reading, was more like the self. This was always followed by a follow-up question asking the student to point to a smaller or larger circle (1.1 and 2.3 cm in diameter, respectively) to indicate “a little” versus “a lot” of similarity. This two-step formulation of each question (known as “branching”; Krosnick and Presser, 2010) was done to keep the number of choices simple and age appropriate (Master et al., 2017a). Positive values indicated choice of the math character as more like the self.

The advantage of using the two-item explicit self-concept measure instead of a longer scale is that it is simple enough to be used in the age group tested. While using two-item measures may seem like an oversimplification of a multidimensional construct such as math self-concept, prior research suggest that such two-item measures are predictive of cognitive and behavioral outcomes in math contexts. For example, research has found that these math self-concept measures are predictive of standardized math test scores (Cvencek et al., 2015). There is also evidence showing that these measures demonstrated theoretically-expected evidence of cognitive–affective consistency or “balance” within child samples (Cvencek et al., 2014). This explicit measure typically exhibits similar internal consistency as six-item multidimensional math self-concept measures. In the current study, the explicit measure was internally consistent, as indicated by both satisfactory Cronbach’s alpha (α = 0.71) as well as a strong correlation between the two items, *r* = 0.55, *p* < 0.001.

### Experimental Intervention Tasks

The interventions were administered individually immediately after the pretest. The order of implicit and explicit interventions was counterbalanced across participants. The entire intervention protocol took approximately 25 min. Each student was initially assigned to one of three groups: math-intervention (treatment), reading-intervention (reverse-treated comparison), or no-intervention group (untreated comparison). In the math-intervention group, students completed four tasks with math-related stimuli. In the reading-intervention comparison group, students completed the tasks with reading-related stimuli. In the no-intervention comparison group, students did not complete any intervention activities, and spent 5–10 min waiting in the same testing room before completing the posttest measures.

#### Intervention Task #1: Activating Positive In-Group Attributes

This intervention task was designed to highlight positive attributes about students’ in-group’s math performance. The rationale for this task was that reminding students about positive stereotypes about groups to which they belong can have positive effects on students’ own identification with mathematics. This activity was designed to activate positive attributes about the math ability of students’ in-group (Ambady et al., 2001). Findings of the Programme for International Student Assessment (PISA) study were used to create a short lesson that was shown to students. Students viewed a series of PowerPoint bar graphs that depicted Madrid students’ math (or reading) performance relative to other Spanish and European students, and showed that Madrid children in general scored higher on math than students did on average in other regions of Spain (e.g., Catalonia, Navarre) or some countries in Europe (e.g., England, Germany).

In the math-intervention (treatment) group, the scores represented in bar charts were described as “scores on a test that measures how good at math you are.” The scores presented were the actual average PISA math scores for Madrid. In the reading-intervention group, the scores represented in bar charts were described as “scores on a test that measures how good at reading you are.” In the reading-intervention (comparison) group, the graphs used were the same ones used in the math-intervention group. Students in the no-intervention (comparison) group did not see any graphs.

#### Intervention Task #2: Expressing *Me* = *Math* Identity

This intervention task aimed to allow students to verbally express positive math self-concepts and reflect on why they are personally important to them. The principle underlying this task was that engaging in self-affirmations—such as seeing oneself as efficacious—can alleviate the stress in achievement contexts by buttressing self-worth in that domain. Such self-affirmations are often induced by having students consciously reflect on personally important values, such as the importance of a self-defining skill. In the current study, the activity was designed to allow students to express and endorse their math (reading) identity and why these activities were important to them, in an adaptation of the intervention developed for older students by Cohen et al. (2006). Students in the math-intervention (treatment) group were asked to answer, “How good at math are you?” on a Likert scale ranging from 1 (*not good at all*) to 7 (*very good*). The experimenter reinforced their response (“It sounds like you are kind of/pretty good at math”) and, most importantly, asked them to “Now write a few reasons why you think you are good at math.” As reported below, children’s ratings of their own math ability were highly positive (all *p*s < 0.001). (Although this adaptation highlighted students’ ability in math, Mueller and Dweck, 1998, the ratings and explanations were all generated by students themselves, which protects against any possible threatening aspects of the evaluation, Yeager and Walton, 2011). The experimenter then discussed their answers with them.

Next, students in the math-intervention (treatment) group were asked, “How important is it for you to get good grades in math?” on a Likert scale ranging from 1 (*not at all important*) to 7 (*very important*). The experimenter reinforced their response (“It sounds like math is kind of/pretty important to you”) and asked them to “Now write a few reasons why you think getting good grades in math is important.” The experimenter then discussed their answers with them. This activity provided students with an opportunity to verbally express positive math self-concepts and self-affirm why being good at math was personally important to them (in line with Cohen et al., 2006). Students in the reading-intervention (comparison) group underwent the same procedures with questions about reading. Students in the no-intervention (comparison) group did not complete the affirmation activity. Responses from students in both the math-intervention and reading-intervention groups were highly positive on the 7-point Likert scale (good at math: *M* = 5.91, *SD* = 1.11; math important: *M* = 6.67, *SD* = 0.99; good at reading: *M* = 5.66, *SD* = 1.17; reading important: *M* = 6.48, *SD* = 0.89). Ratings of ability in math versus reading were not significantly different between groups, *p* = 0.21, nor were ratings of the importance of getting good grades, *p* = 0.26.

#### Intervention Task #3: Approaching Math

This intervention task was designed to allow students to physically “approach” math. The idea behind this task is that people generally evaluate objects and categories more favorably following the performance of approach, as opposed to avoidance, actions, especially when evaluations are measured at an implicit level. Consequently, giving students practice in responding to academic subjects by engaging in approach behaviors, which are known to be related—both semantically and behaviorally—with bringing categories closer to the self, can positively impact students’ orientation to these subjects, at least at an implicit level. This activity was designed to have students associate math with approach (“positive”) behaviors (following a procedure designed for adults by Kawakami et al., 2008). In the math-intervention (treatment) group, students were instructed to pull a joystick toward themselves when presented with math images (i.e., cartoons of children doing math or math objects such as calculators) and to push the joystick away when presented with reading images (i.e., cartoons of children reading or objects such as books). Students in the reading-intervention (comparison) group were instructed to pull the joystick toward themselves for reading images and push it away for math images. Students in the no-intervention (comparison) group did not complete any approach tasks. In both math-intervention and reading-intervention groups, students completed four blocks of 40 trials.

#### Intervention Task #4: Sound Cueing for Identification and Positivity

This intervention task was designed to allow students to hear interesting sound cues linked to math. Prior research has shown that pairing concepts (such as “mathematics”) with subtle auditory cues—which participants have previously been trained to associate with self and positivity—can effectively enhance the implicit *me* = *math* and *math* = *good* linkages. This activity was designed to use sound cues in a task that has been used to reduce implicit gender biases in adults (Hu et al., 2015). Students engaged in two phases of training activities that rely on sound cues to strengthen the *me* = *math* and *math* = *good* associations.

During the first training phase, students viewed several types of image–word pairings but were required to attend and respond only to pairings that involved either (i) *me* pronouns and a *math* image, or (ii) a *good* word and a *math* image. Two attention-getting, frequency-modulated sounds were presented during the first training phase: one after correctly linking math and the self (*me* = *math*) and the other after linking math to something positive (*math* = *good*). Students in the reading-intervention comparison group were instructed to respond only to *me* and *reading* pairings and *good* and *reading* pairings. Students in the no-intervention comparison group did not complete any sound cueing trials. During the first training phase, students in the math-intervention (treatment) group and reading-intervention (comparison) group completed three blocks of 18 trials.

To underscore these associations, students also completed a second training phase. In each of the activities in the second training phase, the same two sounds from the first training phase prompted students to form a corresponding image–word pairing by using a computer mouse to drag the image to the appropriate target word. Students in the no-intervention (comparison) group did not complete any image dragging trials. During the second training phase, students in the math-intervention (treatment) group and reading-intervention (comparison) groups completed three blocks of 18 trials.

### Posttest

Following the administration of the four intervention tasks, and an optional 5–10 min break, all students completed the same implicit and explicit measures of math self-concept (i.e., posttest was administered in the same session as pretest, in the same location/test room, on the same laptop computer, and closely following the interventions, all as recommended by Shadish et al., 2002, for methodological reasons).

### Treatment Fidelity

Three project coordinators supervised nine experimenters from the beginning to the end of the experiment. A 3-step plan for experimenter training was implemented prior to the start of the study. First, each experimenter was provided with the Treatment Manual in Spanish, which described the study protocol in detail. Second, project coordinators carried out training sessions in which they modeled the interventions with each experimenter prior to the start of the study. Third, project coordinators observed experimenters and provided training feedback in the form of group discussions with all experimenters over the course of several weeks, during which specifics of the interventions were repeatedly reviewed. These team meetings focused on evaluating the experimenters’ adherence to the research protocol. The sessions highlighted experimenters’ successes and failures, offered constructive feedback from project coordinators and other experimenters to increase the fidelity of interventions, and clarified procedures to minimize departures from protocol.

Once the study started, experimenters were also observed by their peers (i.e., other experimenters) during the interventions, which provided additional rounds of “real-time” feedback to correct any significant departures from protocol. Treatment fidelity is important to consider, because when interventions fail to produce expected effects, there is potential to conclude (erroneously) that observed results are due to the conceptual or methodological problems with a particular intervention, rather than the fact that it was not delivered as intended (Dusenbury et al., 2003).

In this study, treatment fidelity was quantified in two ways. First, by design, we kept track of intervention duration and compared the average duration for math-intervention (*M* = 23.75 min, *SD* = 2.47 min) and reading-intervention (*M* = 23.14 min, *SD* = 2.24 min) groups. These did not differ in temporal duration (*p* = 0.14), ruling out the possibility that students in the math-intervention group received “more” intervention than the students in the reading-intervention group. Second, any instances of “unforeseen events” that occurred were written down (e.g., school headmaster being present during interventions, bell ringing during intervention administration, student not following all of the instructions, etc.). This occurred for only 10.3% of the students. A Chi-square analysis was conducted to examine whether the number of these unforeseen events varied by experimental group and revealed no statistically significant effects, *p* = 0.46.

### Year-End Math Achievement

We also wanted to examine the degree to which our math-intervention effects might be associated with long-term academic outcomes. We re-contacted the schools toward the end of the academic year (collected in June of both 2016 and 2017 school years) and requested measures of math achievement (i.e., grades in mathematics from the year-end report cards) for students in the math-intervention group (institutional constraints and costs prevented us from requesting year-end report cards from more than about 100 students). We achieved a 57% compliance rate for this aspect of the study, *n* = 56 out of 99 students. No other achievement data was provided by the schools.

## Results

Several preliminary analyses were conducted to check whether demographic factors (gender, age) and classrooms/schools from which children were recruited had significant effects on any of the pretest or posttest results. As expected, none of them did, all *p*s > 0.10. Therefore, the analyses are reported by collapsing across these factors. The results are organized in sections: (i) preliminary analyses, (ii) pre–post change on math self-concepts for treatment versus comparison groups, followed by (iii) analyses evaluating long-term relations between treatment outcomes and end of year math achievement. Table 1 displays correlations among study variables.

**TABLE 1:** Correlations for all implicit and explicit measures.

Measure	1	2	3	4
(1) Implicit MSC (Pretest)	–			
(2) Implicit MSC (Posttest)	0.32***	–		
(3) Explicit MSC (Pretest)	0.21*	0.26**	–	
(4) Explicit MSC (Posttest)	0.23*	0.25**	0.76***	–

MSC, Math Self-Concept; *p < 0.01; **p < 0.001; ***p < 0.0001.

### Main Analyses: Malleability of Self-Concepts

#### Preliminary Analyses

We first checked whether there was any difference in the pretest scores as a function of group. Two one-way analyses of variance (ANOVAs) were performed on math self-concept pretest scores (one for implicit, and one for explicit measures) with experimental group as a between-groups factor. As expected, neither the ChIAT measure of implicit math self-concept nor the students’ verbal report of explicit math self-concept showed a pretest difference as a function of group (implicit, *p* = 0.70; explicit, *p* = 0.25). In addition, we examined whether the two comparison groups (reading-intervention group, *n* = 49, and no-intervention group, *n* = 27) differed on any of the implicit or explicit measures. There were no significant differences (see Supplementary Material). Thus, for the main analyses, we combined the reading-intervention and no-intervention groups into a combined comparison group (*n* = 76), which was compared to the math-intervention treatment group (*n* = 99).

#### Pre–Post Change

One-way analyses of covariance (ANCOVAs) were conducted to examine differences between the treatment and comparison groups on math self-concept posttest scores, controlling for pretest scores. This approach ensures that posttest differences result from the treatment, and not leftover effects of random pretest differences between groups.

##### Implicit measures

**FIGURE 1:**
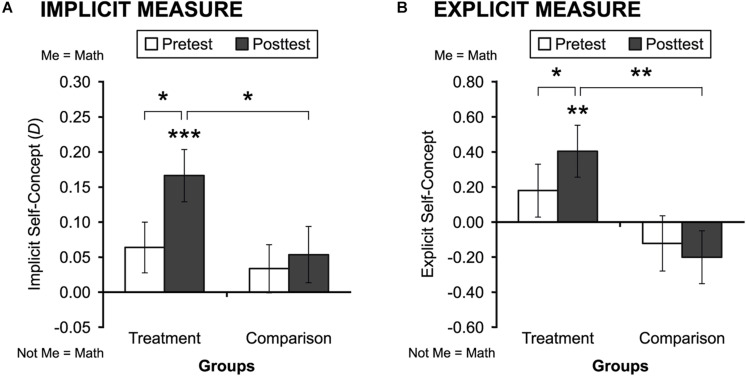
Students’ implicit **(A)** and explicit **(B)** math self-concepts at pre- and posttest, by treatment and comparison group. *N*s are 99 and 76 for treatment and comparison groups, respectively. Error bars show ±1 *SE*. *p < 0.05; **p < 0.01; ***p < 0.001.

The results for the implicit measures are displayed in Figure 1A. On both pre- and posttest implicit math self-concept measures, positive scores indicated *me* = *math* associations. As can be seen from the left two bars in Figure 1A, students in the math self-concept treatment group displayed stronger math self-concepts at posttest than at pretest. In contrast, and as shown in the right two bars in Figure 1A, students in the comparison group showed no significant gain in math self-concepts from pretest to posttest. The ANCOVA for the implicit measures revealed a significant effect of the math intervention on posttest math self-concept *after controlling for pretest*, *F*(1,172) = 3.73, *p* = 0.05, *d* = 0.29. Moreover, as shown in Figure 1A, paired-sample *t*-tests revealed that the pre–post change was significant in the treatment group, *t*(98) = 2.36, *p* = 0.02, *d* = 0.28, but not in the comparison group, *p* = 0.65. As expected, the pretest scores did not differ between the treatment and comparison group, *p* = 0.55; yet, the posttest scores in the treatment group were significantly higher in the *me* = *math* direction than in the comparison group, *t*(173) = 2.03, *p* = 0.044, *d* = 0.31. Finally, we compared the implicit scores to 0 (equally strong math and reading self-concepts), and only the scores in the treatment group at posttest were significantly different from 0, and they were in the *me* = *math* direction (*M* = 0.17, *SD* = 0.38), *t*(98) = 4.41, *p* < 0.001, *d* = 0.44.

##### Explicit measures

The results for the explicit measures are displayed in Figure 1B. On both pre- and posttest explicit math self-concept measures, positive scores indicated *me* = *math* associations (and negative scores indicated *not-me* = *math* associations). As can be seen from the left two bars in Figure 1B, students in the treatment group displayed stronger math self-concepts at posttest than at pretest. In contrast, and as shown in the right two bars in Figure 1B, students in the comparison group displayed no significant gain in math self-concepts from pretest to posttest. The ANCOVA for the explicit measures revealed a significant effect of the math intervention on posttest explicit math self-concept after controlling for pretest, *F*(1,172) = 7.46, *p* = 0.01, *d* = 0.42. Moreover, as shown in Figure 1B, paired-sample *t*-tests revealed that the pre–post change was significant in the treatment group, *t*(98) = 2.46, *p* = 0.016, *d* = 0.15, but not in the comparison group, *p* = 0.53. As expected, the pretest scores did not differ between the treatment and comparison groups, *p* = 0.17; and the posttest explicit scores in the treatment group were significantly higher in the *me* = *math* direction than in the comparison group, *t*(173) = 2.82, *p* = 0.01, *d* = 0.43. Finally, we compared the explicit scores to 0, and only the scores in the treatment group at posttest were significantly different from 0, and they were in the *me* = *math* direction (*M* = 0.40, *SD* = 1.47), *t*(98) = 2.73, *p* = 0.01, *d* = 0.27.

### Analyses of Year-End Math Achievement

We also examined correlations between the pre- and posttest scores and year-end math achievement for the treatment participants (collected approximately 3 months following posttest). Neither the implicit pretest measure nor the explicit pretest measure was correlated with year-end achievement, *p*s > 0.12. For posttest scores, implicit math self-concepts were significantly correlated with year-end achievement, *r*(54) = 0.42, *p* = 0.001, but explicit math self-concepts were not, *p* = 0.83 (see Discussion). Moreover, a partial correlation of posttest implicit math self-concepts remained significant with achievement *after controlling for* pretest implicit math self-concepts, *r*(53) = 0.38, *p* = 0.004.

## Discussion

Third-grade students demonstrated significant gains on math self-concepts following a math self-concept field intervention that took place in a school setting. The intervention involved: (i) highlighting positive attributes about students’ in-group’s math performance, (ii) verbally expressing positive math self-concepts, (iii) physically “approaching” math, and (iv) hearing interesting sound cues linked to math. Students in the math-treatment group demonstrated stronger math self-concepts at posttest after controlling for pretest scores, and the comparison groups did not. This was true using *both* implicit and explicit measures. In addition, implicit math self-concepts in the intervention group at posttest were associated with higher year-end math achievement, assessed approximately 3 months later. This study suggests that a field intervention can be delivered one-on-one to elementary students during school hours.

The positive effect of the math intervention on both implicit and explicit measures is noteworthy, with implicit effects being particularly informative. A well-established criticism of studies that use only explicit, self-report outcome measures is that children may sometimes distort their true beliefs about math when verbally describing them based on what they think the adult wants to hear (“desirability effects”). For this reason, we used both implicit and explicit measures. As shown here, the measures of *implicit* math self-concepts can provide a valuable, even unique, window into investigating emerging beliefs about self and math in young children (see also Meltzoff and Cvencek, 2019).

A question of relevance to developmental and educational science is why this intervention with young children was successful when studies with *adults* have shown that implicit associations are difficult to change (Lai et al., 2014). Two factors might have played a role. First, our intervention was designed to incorporate several features that have been specifically linked to success in changing implicit associations, such as incorporating elements that are highly self-relevant (e.g., self-affirmation, activating positive stereotypes) and using multiple techniques (e.g., approach/avoid behaviors, sound cueing to call attention to positivity) to target self-concepts (Lai et al., 2014). Second, the age tested here may be a period during which these implicit associations are particularly malleable (Lai et al., 2016; Gonzalez et al., 2017; Meltzoff and Cvencek, 2019). Self-concepts in adults have been influenced by many prior experiences which may make them difficult to shift (Lai et al., 2016). In contrast, children’s implicit cognition is based on fewer experiences and may therefore be more open to change (Gonzalez et al., 2017). Relatedly, preschool children or those substantially younger than studied here may be unable to integrate new experiences (Bigler and Liben, 2007). Thus, there may conceivably be a “Goldilocks period” in development (we speculate between ages 8–12; more research needed), in which children’s implicit self-concepts about math are easier to change than adults’ or substantially younger children’s (Vezzali et al., 2012). We also note that we found no effects of gender on pretest or posttest measures. This pattern of results was unsurprising, given that this age group was selected intentionally to involve children before gender differences in math self-concepts have developed (see Cvencek et al., 2011; Master et al., 2017a).

The findings that posttest implicit math self-concept scores were associated with year-end math achievement raises two interesting theoretical questions. The first one involves why pretest math self-concepts were not associated with academic achievement. Math self-concepts of early elementary-school children are, on average, very positive and only weakly, if at all, associated with external indicators such as grades (Ehm et al., 2019). Here, we are able to demonstrate that the theoretically expected relations to academic achievement can be obtained following an intervention, lending further credence to the idea that math self-concepts are malleable and undergoing developmental change during elementary-school years (Cvencek et al., 2011; Weidinger et al., 2018).

A second question concerns why implicit self-concepts, but not explicit self-concepts, were linked to achievement. Several possibilities bear consideration. On the one hand, other research has also found that in many cases implicit measures predict behavior and achievement better than self-reports (Rudman, 2004; Cvencek et al., 2015; see also Greenwald et al., 2009), and it is possible that social desirability of giving the answer the experimenter wants may add noise to the results when using explicit measures alone as outcomes. On the other hand, there are measurement differences between implicit and explicit measures: The explicit self-concepts were measured on a Likert scale, and the implicit self-concepts were measured on a continuous scale. Previous research has shown that relatively coarse Likert scales can cause information loss and reduce the probability of detecting true effects (Russell and Bobko, 1992; see also Albaum et al., 1981, and Wu and Leung, 2017). Because implicit measures allowed for finer assessment of individual differences than the 4-step Likert scales used in the current study, they may have been more sensitive to pre–post treatment changes at an individual level.

These findings showing a positive association between the interventions and the year-end math outcomes also call for a discussion about possible mediating mechanisms. Based on the current feasibility study we speculate that experiences, such as those provided by the interventions used here, may enhance the implicit *me* = *math* linkages, which could have downstream, cascading consequences for motivation, such as putting in extra effort or persistence on math activities or approaching (rather than avoiding) math-related endeavors. These approach/persistence/motivation behaviors could in turn provide learning experiences that build math skills and thus affect year-end achievement through a positive recursive cycle (Cohen et al., 2006; Master and Meltzoff, 2020), in which stronger implicit math self-concepts lead to higher achievement, which then reinforces positive self-concepts. Based on the current findings, future research should examine how variations in frequency of such brief interventions (daily? weekly? quarterly?) map on to educational outcomes that matter for children, which will have implications for how these interventions could potentially be used in practice.

### Limitations, Lessons Learned, and Future Research

The reported work has several strengths but is not without limitations. First, we acknowledge the nonrandom assignment of participants to treatment and comparison groups. Importantly, the treatment and comparison groups did not differ at pretest for either implicit or explicit measure, thus reducing concerns that the groups differed in important ways before the interventions were initiated. A critical direction for future research is to replicate this study with a large, pre-registered, randomized controlled trial (RCT) educational intervention.

A second limitation concerns the follow-up on math achievement. Math grades used in this research corresponded to the teachers’ ratings. As such, they may be influenced by subjectivity, and also capture other aspects of student learning, such as effort, classroom behavior, or the relationship with the teacher (McMillan et al., 2002). The use of a standardized math test would be more informative insofar as it would permit testing directly whether the students with higher levels of math self-concept were those with the better mathematical performance. In addition, we were able to obtain year-end achievement data only for the treatment (math-intervention) group, and had 57% compliance in obtaining the year-end math achievement scores. This was due to the constraints of working with this particular school district. The use of a standardized math test with all students, especially at the school-level, would permit evaluating a direct effect of treatment condition on achievement by comparing the average achievement for the subsample that was intervened on to the average achievement of another sample of students in the same school and grade that was not intervened on. Future work should initiate randomized sampling from both treatment and comparison groups, coupled with procedures or compensation that might encourage higher compliance. However, even under our limited conditions, and with a modest sample size, we obtained a medium-sized relation between students’ enhanced posttest implicit math self-concepts and their higher year-end math achievement (effect size of *r* = 0.42). A direction for future research is to examine the relations to math achievement with repeated assessments throughout the academic year. The current study’s intervention was relatively brief, approximately 25 min total. What happened in a brief, one-shot intervention does not guarantee long-term effectiveness. Evaluating the effects of the intervention after a longer delay will allow for stronger inferences about the durability of the effects, which are important for both practical and theoretical concerns.

Third, this quasi-experimental study was primarily concerned with assessing the *feasibility* of whether an intervention combining explicit and implicit approaches has potential in authentic school settings. In doing so, we did not control for covariates that could account for differences between the treatment and comparison group. For example, family SES, parental education, math curriculum used in school, and teachers’ experience could all contribute to the development of children’s math self-concepts. Given that this study was not an RCT, we acknowledge that these factors were notnecessarily random across conditions. Our hope is that the positive results from this feasibility study, including the lessons learned (see below), might spark future work using similar techniques and adopting a gold-standard, RCT methodology, which will deal effectively with unknown and unmeasured environmental covariates.

Fourth, implicit measures by design involve *relative* comparison between two contrasting target categories (Greenwald et al., 2009). Implicit measures that contrast math with reading/language are common in research about academic topics with adults (Nosek et al., 2002; Nosek and Smyth, 2011) and children (Cvencek et al., 2011, 2015). At the same time, the relative nature of implicit measures makes it difficult to conclude whether the current intervention: (i) only enhanced students’ math self-concepts or (ii) enhanced math self-concepts while also weakening reading self-concepts (which would be in line with the so-called *ipsative self-concept hypothesis*, according to which, as self-concept in one domain goes up (e.g., math), self-concept in other domains (e.g., verbal) should go down; Parker et al., 2015; Umarji et al., 2018). While the relative implicit measures do not allow us to distinguish between these two alternatives, they are still useful in evaluating the effectiveness of interventions aimed at improving math outcomes, inasmuch as they have been found to be positively related to both absolute math achievement (e.g., performance on a standardized math test; Cvencek et al., 2015), as well as relative math achievement (e.g., SAT math minus verbal difference; Nosek and Smyth, 2011).

Fifth, this study used multiple components to target self-concepts (four tasks), and this limits our ability to specify the precise factor(s) that may be most important. Our rationale was that a multicomponent approach designed to boost both explicit and implicit math self-concepts might be more beneficial than a more narrowly designed intervention. But using multiple components does not allow us to determine which specific elements were necessary and/or sufficient, which would be needed to address mediating mechanisms. It would be useful for future interventions to test each intervention component separately.

Finally, more work is needed to make these math interventions culturally appropriate for other Spanish-speaking student groups, including Hispanic/Latinx students in the United States. Such students in the United States commonly experience a number of social, cultural, and economic barriers that affect their academic achievement, from poverty to issues of ethnic-racial discrimination. While these are not shared by Spanish children living in Madrid, the materials and activities adapted here provide a first step toward actionable practices in the Spanish language that can be used in future work with Spanish-speaking students in other countries beyond Spain. We acknowledge too that the cultural setting matters in this research, as in all work on the education of our children (e.g., Lee et al., 2020). Thus, “what works” in Madrid will not necessarily directly transfer to other cultures—even to other Spanish-speaking cultures inasmuch as different countries, regions, and school systems may well have different needs, practices, and sociocultural norms. More broadly, future interventions aimed at enhancing students’ math self-concepts should not only take into account the sociocultural character of students’ math self-concepts, but also the dynamic character of students’ self-concepts as well as how dominant sociocultural practices interact with students’ self-concepts.

There were also three salient lessons learned from this study, which should be considered when designing future interventions to enhance students’ math self-concepts. First, we demonstrated the feasibility of combining four tasks into one session in an age-appropriate manner. Children understood the directions and they were able to complete the protocol; but there would need to be adjustments to make the procedures developmentally appropriate at different ages. Second, we were able to implement the protocol within a school setting, asking the students to leave the classroom for an acceptable short duration, and we were able to monitor treatment fidelity quite closely, which standardized the protocol. We believe that monitoring treatment fidelity was an important aspect of this research. Third, we showed that the effects of multicomponent treatment can be measured using pre–post change in elementary school, and our procedures were enjoyable to the children at the age tested, which is important to ensure that children stay “on task” throughout the session.

### Broader Educational Implications

The current results have potential implications for educational efforts aimed at promoting equity in math achievement. In Spain, mathematics is rated as an important educational area, but beyond Spain a growing number of educators are emphasizing the need to customize individual learning based on students’ personal and academic readiness from Grade 3 onward (Gutiérrez and Rogoff, 2003; Paz-Albo, 2017; Nasir et al., 2020). After further development and formal RCT testing, the type of interventions developed here might prove useful in elementary education as a way to enhance students’ interest and identification with math, and thus their interest and engagement in choosing STEM classes, after-school activities, and summer camps, and to influence their career aspirations. Moreover, the technology-based nature of the intervention provides the opportunity of expanding and refining this work so that it could be incorporated into online learning software.

The findings also have potential implications for the choices that administrators and researchers make about tutoring and interventions for math. Some interventions designed to improve children’s counting competencies have been shown to be highly effective (Clements and Sarama, 2011), but they are also known to be somewhat time-consuming and costly. It has also been recognized that endeavoring to enhance students’ beliefs and identifications with math (so-called “non-academic factors”) is desirable because this may be less expensive and (possibly) more enjoyable for the students. Indeed, previous work with middle-school students has been able to show that non-academic interventions, such as mentoring students about the malleability of “intelligence,” can boost standardized math test scores (Good et al., 2003; see also Dweck, 2006; Blackwell et al., 2007). Taking all this together, it would seem judicious for future work to *combine* programs aimed at improving math instruction and math skill development (e.g., Clements et al., 2020) with the types of social–cognitive interventions used in our current work as well as those of others (Yeager and Walton, 2011; Rhodes et al., 2019; Master and Meltzoff, 2020). This might allow us to assess whether a more comprehensive intervention strategy would be even more effective or longer lasting than any of the approaches listed above taken in isolation.

## Conclusion

This study tested the feasibility of whether combining explicit and implicit approaches into a multicomponent intervention to help children form more positive beliefs about themselves and math can be administered in an authentic school setting. The results suggest that a field intervention can be delivered one-on-one to elementary students during school hours. The intervention was found to be effective insofar as third-grade students demonstrated significant gains on math self-concepts following the intervention. After further development, the novel intervention utilized here—or similar child-friendly ones—might have practical use for helping to spark young students’ self-concepts, interest, and choices around mathematics. It is known, for example, that some elementary-school students decide that they are not “a math person,” and thereafter dis-identify with math as they progress through school (Heyman, 2008; Musu-Gillette et al., 2015). Early interventions may hold promise for preventing or ameliorating this trajectory (Liben, 2015; Master et al., 2017b; Fredricks et al., 2018). More collaborative work between educators and researchers is needed to explore the ways in which interventions may enhance children’s beliefs and attitudes about math over time and in school settings, and also to assess the feasibility of embedding these in the elementary-school curriculum. Well-timed interventions could help ensure that students stay identified with math and have a positive math self-concept very early in the pipeline. When students believe that “math is for me,” it could potentially open the door to a positive relationship with math that will be helpful for broader academic success.

## Data Availability Statement

The dataset generated for this study is available on request to the corresponding author.

## Ethics Statement

The studies involving human participants were reviewed and approved by Universidad Rey Juan Carlos Ethics Committee, approval number 22/2015 and ENM 22/20150712201600317. Written informed consent to participate in this study was provided by the participants’ legal guardian/next of kin.

## Author Contributions

DC, ANM, JP-A, CVHL, and AH-E designed the study and organized data collection. DC analyzed the data. DC, ANM, AM, and JP-A wrote the manuscript. All authors contributed to the article and approved the submitted version.

## Conflict of Interest

The authors declare that the research was conducted in the absence of any commercial or financial relationships that could be construed as a potential conflict of interest.
