# Supplementary material for: Math Is for Me: A Field Intervention to Strengthen Math Self-Concepts in Spanish-Speaking 3rd Grade Children
Source: Front Psychol. 2020 Nov 23;11:593995. doi: 10.3389/fpsyg.2020.593995 (PMC7732437; doi:10.3389/fpsyg.2020.593995)
Supplement: Supplementary file 1 [file Presentation_1.pdf]

Supplementary Material

Math Is for Me: A Field Intervention to Strengthen Math Self-Concepts in Spanish-Speaking 3rd Grade Children

Cvencek, Paz-Albo, Master, Herranz Llácer, Hervás-Escobar, and Meltzoff

Frontiers in Psychology, 2020. doi:10.3389/fpsyg.2020.593995

1 THEORETICAL CONCEPTIONS OF MATH SELF-CONCEPTS

Math self-concepts refer to how children think of themselves in relation to math. As noted in the main text, math self-concepts can be measured in many ways and at many levels (Gunderson et al., 2012). This section provides a more detailed consideration of what is meant by the three "levels," along with examples of the items/measures used to assess them.

At the simple level are *straightforward self-perceptions and identities* such as "the association between *self* and *math*" (Cvencek et al., 2011, p. 766) or students' "evaluative judgments of attributes in discrete domains, such as [math]" (Harter, 2006, p. 509). These simple conceptions are assessed by measuring how quickly students can sort math related words (e.g., *count*, *numbers*) along with self-referring pronouns (e.g., *me*, *I*) in a computerized task in which both *math* and *me* categories require the same response button (as in the ChIAT), or having students indicate similarity with another child who is "good at numbers" (using options from '*1 = A little like*' to '*2 = A lot like*').

At the intermediate level of complexity are cognitively based, self-reflective views of math self-concepts that involve *social comparison* and *future expectancies* such as "children's beliefs about their competence [in math] and how well they expect to perform [in math] in the future" (Simpkins et al., 2012, p. 1021), or "individuals' knowledge and perceptions about themselves in [math] achievement situations" (Bong & Skaalvik, 2003, p. 6). These intermediate conceptions are assessed by having students answer questions such as "How good would you be at learning something new in math?" (using options from '*1 = not very good*' to '*7 = very good*'), or expressing their agreement/disagreement with items such as "Mathematics is one of my best subjects" (using options from '*1 = false*' to '*7 = true*').

At a more complex level are multidimensional models of math self-concepts as *more global, integrative schemas* informed by students' broader academic environment such as "a person's self-perceptions formed through experience with and interpretations of his/her environment… [including] feelings of self-confidence, self-worth, self-acceptance, competence, and ability" (Marsh et al., 2019, p. 333; see also Shavelson et al., 1976), or student's "composite view of him or herself as a [math] student, a view formed through experience and feedback from others… [focusing] primarily on the feelings of self-worth associated with being a [math] student" (Pajares, 2001, p. 29). These more complex conceptions are usually assessed by having students answer questions such as "If the math teacher asks a question, I usually know the right answer" (using options from '*1 = true*' to '*5 = absolutely true*'), or expressing agreement/disagreement with items such as "Compared to others my age, I am good at math" (using options from '*1 = definitely false*' to '*6 = definitely true*').

2 PRELIMINARY ANALYSES

In addition to the analyses reported in the main text, we examined whether the two comparison groups (reading-intervention, n = 49, and no-intervention, n = 27) differed on the implicit or explicit measures. Table S1 provides the means for each group. A 2 (comparison group: reading- or no-intervention) × 2 (measure: implicit or explicit) × 2 (time: pretest or posttest) mixed-model analysis of variance (ANOVA) was performed on math self-concept scores with comparison group as a between-subjects factor and measure and time as within-subject factors. As expected, the main effect of comparison group was not statistically significant, p = .38. These preliminary analyses suggested that the untreated (no-intervention) and reverse-treated (reading-intervention) groups are best combined into one comparison group to increase statistical power. This yielded reasonably large sample sizes in treatment (n = 99) and comparison (n = 76) groups, and allowed for sufficient statistical power to detect even a small positive effect of the math intervention (all statistical comparisons groups reported in the main text had conventionally "small" effect sizes of Cohen's d ranging between .15–.44).

Table S1 | Means and standard deviations for all implicit and explicit measures by each of the three groups.

	Math intervention			Reading intervention			No intervention		
Measure	*N*	*M*	*SD*	*N*	*M*	*SD*	*N*	*M*	*SD*
Implicit MSC (Pretest)	99	0.06	0.36	49	0.05	0.28	27	0.002	0.33
Implicit MSC (Posttest)	99	0.17***	0.38	49	0.06	0.34	27	0.04	0.37
Explicit MSC (Pretest)	99	0.18	1.50	49	-0.24	1.39	27	0.10	1.36
Explicit MSC (Posttest)	99	0.40_a**	1.47	49	-0.30_a	1.36	27	-0.02	1.22

*MSC, Math Self-Concept. Means in a row sharing a subscript are significantly different at p < .01. Asterisks indicate significant difference from 0; **p < .01; ***p < .0001.*

3 DISCUSSION

A question arises as to why students in the math-intervention group showed an increase in math self-concepts, but students in the reading-intervention (a reverse-treated comparison) did not show an increase in reading self-concepts (see Table S1). We do not have a firm answer, but one possibility is that the development of reading self-concepts may follow a somewhat different developmental trajectory than math self-concepts, and this was not the optimal time window for such an intervention. Another (not mutually exclusive) possibility, which we favor, is that math self-concepts are particularly salient and thus amenable to input and change at this age. Math is typically viewed as more challenging than reading (Gunderson et al., 2017), with children reporting that success in math derives more from "natural talent" than effort (Meyer et al., 2015). If students are more concerned that they do not have the ability to succeed in math, then they may be *particularly attentive* to information about math, such as that delivered in the intervention.

4 REFERENCES

Bong, M., and Skaalvik, E. M. (2003). Academic self-concept and self-efficacy: how different are they really? *Edu. Psychol. Rev.* 15, 1–40. doi: 10.1023/A:1021302408382

Cvencek, D., Meltzoff, A. N., and Greenwald, A. G. (2011). Math–gender stereotypes in elementary school children. *Child Dev.* 82, 766–779. doi: 10.1111/j.1467-8624.2010.01529.x

Gunderson, E. A., Hamdan, N., Sorhagen, N. S., and D’Esterre, A. P. (2017). Who needs innate ability to succeed in math and literacy? Academic-domain-specific theories of intelligence about peers versus adults. *Dev. Psychol*. 53, 1188–1205. doi: 10.1037/dev0000282

Harter, S. (2006). “The self,” in *Handbook of Child Psychology: Social, Emotional, and Personality development*, eds N. Eisenberg, W. Damon, and R. M. Lerner, (Hoboken, NJ: Wiley), 505–570.

Marsh, H. W., Pekrun, R., Parker, P. D., Murayama, K., Guo, J., Dicke, T., et al. (2019). The murky distinction between self-concept and self-efficacy: beware of lurking jingle-jangle fallacies. *J. Educ. Psychol*. 111, 331–353. doi: 10.1037/edu0000281

Meyer, M., Cimpian, A., and Leslie, S.-J. (2015). Women are underrepresented in fields where success is believed to require brilliance. *Front. Psychol*. 6, 235. doi: 10.3389/fpsyg.2015.00235

Pajares, F. (2001). Toward a positive psychology of academic motivation. *J. Edu. Res.* 95, 27–35. doi: 10.1080/00220670109598780

Shavelson, R. J., Hubner, J. J., and Stanton, G. C. (1976). Self-concept: validation of construct interpretations. *Rev. Edu. Res.* 46, 407–441. doi: 10.3102/00346543046003407

Simpkins, S. D., Fredricks, J. A., and Eccles, J. S. (2012). Charting the Eccles’ expectancy-value model from mothers’ beliefs in childhood to youths’ activities in adolescence. *Dev. Psychol.* 48, 1019–1032. doi: 10.1037/a0027468
